# Significant role and the underly mechanism of cullin-1 in chronic obstructive pulmonary disease

**DOI:** 10.1515/med-2024-1070

**Published:** 2024-11-18

**Authors:** Wenbo Hao, Fei Lin, Weili Kong, Hanbing Shi, Haiying Dong, Zhanjiang Guan, Guohua Liu, Xiao Wang, Li Wang, Moran Liu, Yunfei Jiang

**Affiliations:** Cardiothoracic Surgery, The Third Affiliated Hospital of Qiqihar Medical University, Qiqihar, 161006, China; Endocrinology, The Third Affiliated Hospital of Qiqihar Medical University, Qiqihar, 161006, China; Department of Respiratory and Critical Care Medicine, The Third Affiliated Hospital of Qiqihar Medical University, Qiqihar, 161006, China; Pathology and Pathophysiology, Qiqihar Medical University, Qiqihar, 161006, China; Intensive Care Unit, The Third Affiliated Hospital of Qiqihar Medical University, Qiqihar, 161006, China; Radiology Imaging Diagnosis Center, The Third Affiliated Hospital of Qiqihar Medical University, Qiqihar, 161006, China; Test Center, The Third Affiliated Hospital of Qiqihar Medical University, Qiqihar, 161006, China; Department of Respiratory and Critical Care Medicine, The Third Affiliated Hospital of Qiqihar Medical University, No. 27 Taishun Street, Tiefeng District, Qiqihar, 161006, China

**Keywords:** chronic obstructive pulmonary disease, cigarette smoke extract, cigarette smoke inhalation, cullin-1, p53 pathway

## Abstract

**Background:**

This study investigated the role and mechanisms of cullin-1 (CUL1) in chronic obstructive pulmonary disease (COPD).

**Methods:**

Cigarette smoke extract (CSE)-treated mouse pulmonary microvascular endothelial cells (mPMECs) and cigarette smoke inhalation (CSI)-stimulated mice were used to construct *in vitro* and *in vivo* COPD models, respectively. CUL1 expression was assessed using reverse transcriptase-quantitative polymerase chain reaction, Western blotting, and immunohistochemistry. The 3-(4,5-dimethylthiazol-2-yl)-2,5-diphenyltetrazolium bromide assay and flow cytometry were used to detect cell viability and apoptosis, respectively. We conducted an enzyme-linked immunosorbent assay on mPMECs and bronchoalveolar lavage fluid (BALF) to detect inflammatory factors. Reactive oxygen species, malondialdehyde, and superoxide dismutase were detected using the corresponding kits. The histological characteristics of the lung tissues were determined by hematoxylin and eosin staining.

**Results:**

CUL1 expression was downregulated in COPD. CUL1 overexpression significantly promoted cell viability, reduced cell apoptosis, and inhibited inflammatory responses and oxidative stress in CSE-treated mPMECs. These changes were reversed by the p53 agonist nutlin-3. In addition, CUL1 overexpression significantly relieved COPD in mice, as confirmed by the reduced secretion of inflammatory factors in BALF, inhibited oxidative stress response, and improved lung function.

**Conclusion:**

CUL1 plays a protective role in CSE-treated mPMECs and CSI-stimulated mice by inhibiting the p53 signaling pathway.

## 1 Introduction

Chronic obstructive pulmonary disease (COPD) is a common chronic respiratory disease characterized by irreversible obstructive ventilation dysfunction. It is currently the third leading cause of death owing to respiratory failure and severe complications worldwide, placing an enormous burden on the global economy and health [1,2]. In 2019, 3.23 million people died of COPD worldwide. As the population ages, the number of deaths from COPD and its complications will continue to increase, showing a more severe trend over time [3]. In recent years, a preference for early prevention and disease stabilization of COPD has emerged. Studies have shown that smoking cessation, avoiding exposure to polluted environments, and exercising regularly can help alleviate symptoms [4,5,6]. In addition, medication has remained the primary treatment option, with options including long-acting β2 agonists (LABAs), inhaled corticosteroids (ICS), and long-acting muscarinic antagonists (LAMAs) depending on patient response and preference [4]; in particular, the combination of LABA and LAMA is more effective [7]. Furthermore, a series of molecular-targeted therapeutic drugs have been developed to prevent and control COPD by targeting molecules involved in signal transduction. Studies have shown that resveratrol, a sirtuin 1 agonist, can reduce lung inflammation in rats with COPD, making it a potential drug candidate for the treatment of COPD [8]. Reports have stated that troxerutin can inhibit the occurrence and development of COPD and may become an important drug for the treatment of COPD in the future [9,10].

Cullin-1 (CUL1), a member of the cullin ubiquitin ligase family, is involved in the process of ubiquitination. CUL1, together with the adaptor proteins S-phase kinase-associated protein 1 (Skp1) and RING-box protein 1 (RBX1), forms the Skp1–Cullin–F-box (SCF) complex [11]. It regulates various biological processes such as cell development, differentiation, and death by binding to F-box proteins [12]. CUL1 has been suggested to play a role in embryonic development [13]. Zhang et al. reported that abnormal expression of CUL1 may be associated with preeclampsia [14]. Sun et al. reported that aberrant CUL1 ubiquitin modification was involved in the pathogenesis of recurrent spontaneous abortion [15]. In addition, CUL1 was shown to promote fibroblast proliferation in patients with COPD by mediating functional signals of organelle fission, thereby participating in disease progression [16]. However, the potential relationship between the function, pathways, and physiological processes of CUL1 in COPD has not been clearly elucidated. Therefore, this study explores the specific role and potential molecular regulatory mechanisms of CUL1 in COPD.

The aim of this study was to investigate the role of CUL1 in cigarette smoke extract (CSE)-induced human pulmonary microvascular endothelial cell injury and in cigarette smoke inhalation (CSI)-stimulated mice as well as to analyze the underlying regulatory molecular mechanisms, thereby providing a potential treatment strategy for COPD.

## 2 Methods and materials

### 2.1 Reagent

CSE purchased from PythonBio (AAPR551, Guangzhou, China) was used for the construction of the COPD model.

### 2.2 Clinical sample collection

Serum samples were collected from 20 patients with COPD and 20 healthy volunteers. Patients who had received treatment were excluded from the study. This study was approved by the Ethics Committee of Qiqihar Medical University (Approval no. [2021]51), and all patients who participated in the study provided written informed consent.

### 2.3 Mouse pulmonary microvascular endothelial cells (mPMECs) culture

mPMECs were obtained from Procell (cat. no. CP-M001; Wuhan, China). The mPMECs were cultured in a mouse pulmonary vascular endothelial cell culture medium (cat. no. CM-M001; Wuhan, China).

### 2.4 Animals

Twenty-six-week-old male C57BL/6J mice were purchased from the Experimental Animal Department, Harbin Medical University (Harbin, China). The mice were randomly divided into four groups: control, model, model + control-plasmid, and model + CUL-plasmid. All mice were kept at standard conditions (20–25°C, 30–50% relative humidity, and a light/dark cycle of 12 h) and had access to food and water ad libitum.

### 2.5 Mice model of COPD induced by cigarette smoke

The COPD mouse model was established as previously described [17]. Briefly, the mice were placed in an airtight box and received eight cigarettes twice daily for 30 min each time, with a smoke-free interval of 3 h for the first 2 weeks. Fifteen cigarettes were subsequently administered twice daily for 30 min each time, with a smoke-free interval of 3 h for the next 10 weeks.

### 2.6 Reverse transcriptase quantitative polymerase chain reaction assay

After treatment, according to the instructions, the RNA was first extracted from the cells and tissues using TRIpure Total RNA Extraction Reagent (EP013, ELK Biotechnology, Wuhan, China) and then reverse transcribed into cDNA using EntiLink™ 1st Strand cDNA Synthesis Super Mix (EQ031, ELK Biotechnology, Wuhan, China). The quantitative real-time reverse transcription PCR (qRT-PCR) experiments were performed using the EnTurbo™ SYBR Green PCR SuperMix (EQ001, ELK Biotechnology, Wuhan, China). The 2^−ΔΔCt^ method [18] was used to detect the expression levels of CUL1 and glyceraldehyde 3-phosphate dehydrogenase (GAPDH). The primer sequences were as follows:

GAPDH:

5′-TGAAGGGTGGAGCCAAAAG-3′ (forward (F)).

5′-AGTCTTCTGGGTGGCAGTGAT-3′ (reverse (R)).

CUL1:

5′-CAATGTTGATGAGGTGGAATTG-3′ (F).

5′-CCTTCCTCATTTTCATGATTCTC-3′ (R).

### 2.7 Western blot assay

The transfected mPMECs were lysed in radioimmunoprecipitation assay buffer (AS1004, ASPEN) for 30 min on ice. The proteins were separated using 10% sodium dodecyl-sulfate polyacrylamide gel electrophoresis, transferred to a polyvinylidene fluoride (PVDF) membrane (IPVH00010, Millipore), and blocked using phosphate-buffered saline with Tween 20 (PBST) (Univ, Shanghai, China) and 5% skim milk powder (AS1033;s ASPEN) for 2 h to avoid nonspecific binding. The PVDF membrane was subsequently incubated with primary antibodies against CUL1 (ab75817, 1:1,000; Abcam), p53 (10442-1-AP, 1:2,000; Wuhan Sanying Biotechnology), p21 (ab109199, 1:1,000; Abcam), caspase-3 (DF6020, 1:500; Affbiotech), caspase-9 (AF6348, 1:500; Affbiotech), and Bcl-2-associated X protein (Bax) (50599-2-Ig, 1:2,000; Wuhan Sanying Biotechnology). After incubating at 4°C for 12 h, the PVDF membrane was washed with PBST and incubated with a secondary antibody (AS1107, 1:10,000; ASPEN) for 2 h, and protein signals were detected using the enhanced chemiluminescence method according to the instructions.

### 2.8 Cell transfection

Control-plasmid and CUL1-plasmid were transfected into mPMECs using jetPRIME reagent (Polyplus, France) according to the instructions of the manufacturer. After treatment with CSE, mPMECs were cultured at 37°C and 5% carbon dioxide (CO_2_) for 48 h. The mRNA level of CUL1 was detected using qRT-PCR. In addition, the protein level of CUL1 was detected using a Western blot assay.

### 2.9 3-(4,5-Dimethylthiazol-2-yl)-2,5-diphenyltetrazolium bromide (MTT) assay

After cell transfection and treatment with CSE, mPMECs were seeded into 96-well plates, and 10 μL of MTT (Beyotime, Shanghai, China) solution was added to the plate. The mPMECs were cultured at 37°C and 5% CO_2_ for 4 h. The supernatant was subsequently discarded and 100 μL of dimethyl sulfoxide solution was added to each well. The cells were allowed to decompose in the dark, and the optical density value was determined at a wavelength of 570 nm using a UV spectrophotometer (Implen nanophoto-330-31) after lysis in the dark.

### 2.10 Flow cytometry assay

The mPMECs were collected after transfection and treatment with CSE, and apoptotic cells were detected using an annexin V and propidium iodide (PI) apoptosis detection kit (Beyotime, Shanghai, China). Cells were pooled and incubated in the dark at room temperature for 20 min, followed by the detection of apoptotic cells using flow cytometry (Beckman Coulter) and analysis of apoptotic cells using the Kaluza analysis software.

### 2.11 Caspase-3 activity assay

To detect the activity of caspase-3 in cells, the transfected cells were digested with trypsin (Gibco, NY, USA), the lysate was added to the sample for 20 min, and the sample was centrifuged at 10,000 rpm for 10 min. The results were analyzed using a microplate reader (Tecan/Infinite F50).

### 2.12 Reactive oxygen species (ROS), malondialdehyde (MDA), and superoxide dismutase (SOD) activity assays

The levels of ROS in the treated cells and lung tissues were detected using a ROS assay kit (E004-1, Nanjing Jiancheng Bioengineering Institute, Nanjing, China) according to the instructions of the manufacturer, and the absorbance was measured at a wavelength of 525 nm using a spectrophotometer (Thermo, USA).

A lipid peroxidation MDA assay kit (A003-1, Nanjing Jiancheng Bioengineering Institute, Nanjing, China) was used to detect the levels of MDA in the treated cells and lung tissues. The assay was performed according to the instructions of the manufacturer, and the absorbance was measured at a wavelength of 532 nm using a microplate reader (Tecan, Switzerland).

A SOD activity assay kit (A001-3, Nanjing Jiancheng Bioengineering Institute, Nanjing, China) was used to detect the levels of SOD in the treated cells and lung tissues. The assay was performed according to the instructions of the manufacturer, and the absorbance was measured at a wavelength of 450 nm using a microplate reader (Tecan, Switzerland).

### 2.13 Enzyme-linked immunosorbent assay (ELISA)

The secretion of interleukin-6 (IL-6), interleukin-1β (IL-1β), interleukin-13 (IL-13), matrix metallopeptidase-12 (MMP-12), and tumor necrosis factor-α (TNF-α) in the treated cell supernatant and bronchoalveolar lavage fluid (BALF) was detected using ELISA kits (ELK Biotechnology, Wuhan, China) according to the instructions of the manufacturer.

### 2.14 Histological examinations

After 24 h of fixation in 4% paraformaldehyde solution, lung tissues were embedded in paraffin and cut into 4 µm slices. The sections were then stained with hematoxylin and eosin (H&E, H9627-25G, Sigma) to observe morphological changes in the lung tissue and photographed using a light microscope (Olympus Corporation, Tokyo, Japan). Mean alveolar septation (mean linear intercept) and number of alveoli per unit area (mean alveolar number) were determined using the counting tool of the Adobe Photoshop CC software.

### 2.15 Measurement of pulmonary function

The Buxco non-invasive animal airway detection system (NAM, Wilmington, DE, USA) was used to measure the lung function of rats. The rats were placed in the non-invasive animal airway detection chamber. Changes in the volume of the plesiogram chamber as the animal breathes were recorded by a screen-type respiratory sensor inside the chamber, amplified by an amplifier, and processed by a computer. Finally, peak inspiratory flow (PIF), peak expiratory flow (PEF), and expiratory flow at 50% ventilation (EF_50_) were determined.

### 2.16 Immunohistochemistry staining

Lung tissues were formalin-fixed, embedded in paraffin, and deparaffinized before incubation in hydrogen peroxide (H_2_O_2_) solution for 15 min to block endogenous peroxidases. The sections were then incubated with rabbit anti-rat CUL1 antibody (1:400, ab202555; Abcam) overnight at 4°C and with goat anti-rabbit secondary antibody (1:200, AS1107; ASPEN) for 1 h at room temperature. Subsequently, the nuclei were identified using 3,3′-diaminobenzidine staining and hematoxylin counterstaining. The sections were then imaged using an Olympus microscope (Olympus Optical Co., Ltd., Japan).

### 2.17 Statistical analysis

The average ± standard deviation measures data from triple repeat. The Student’s *t*-test was used to compare two cohorts. Tukey’s multiple comparison test was used to compare multiple groups. Statistical significance was set at *P* < 0.05.


**Informed consent:** All patients who participated in this study have signed the informed consent.
**Ethical approval:** This study was completed by the Ethics Committee of the Qiqihar Medical University (Approval no. [2021]51).

## 3 Results

### 3.1 CUL1 expression was low in patients with COPD

**Figure 1:**
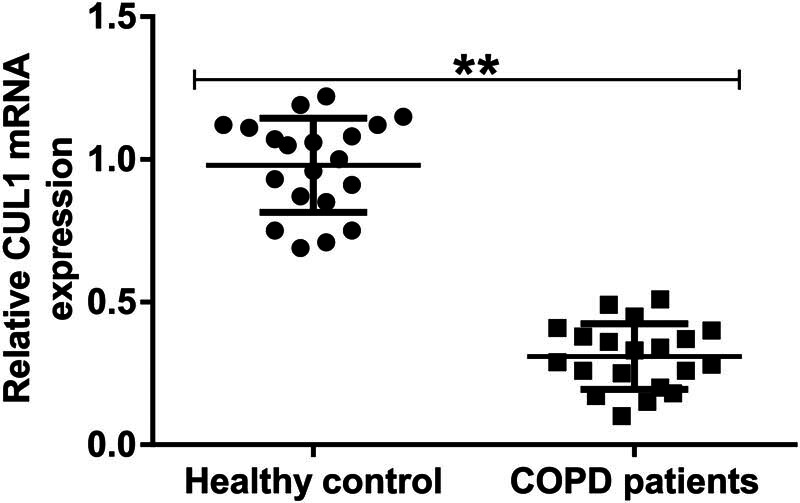
The expression level of CUL1 in the serum of patients with COPD. The expression of CUL1 was detected using qRT-PCR. **P < 0.01. COPD, chronic obstructive pulmonary disease; CUL1, cullin-1; qRT-PCR, quantitative real-time reverse-transcription PCR.

qRT-PCR was performed to investigate the relationship between CUL1 expression and COPD. The expression of CUL1 in the serum of patients with COPD was significantly lower than that in the serum of healthy volunteers (Figure 1), indicating that CUL1 showed low expression in COPD.

### 3.2 CUL1 expression was low in CSE-induced mPMECs

**Figure 2:**
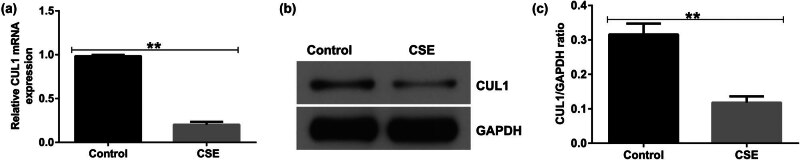
The expression level of CUL1 in a CSE-induced cell model. (a) mRNA expression level of CUL1 detected by qRT-PCR. (b) Protein expression level of CUL1 detected by Western blot assay. (c) CUL1/GAPDH ratio. **P < 0.01. CSE, cigarette smoke extract; CUL1, cullin-1; GAPDH, glyceraldehyde 3-phosphate dehydrogenase; qRT-PCR, quantitative real-time reverse-transcription PCR.

To investigate the role of CUL1 in COPD further, mPMECs were treated with 1% CSE for 24 h to generate a CSE-induced COPD cell model. The results of qRT-PCR and Western blot assays showed that, compared with the control, 1% CSE significantly reduced the expression of CUL1 in mPMECs (Figure 2a–c), and the cell model was successfully constructed for subsequent experiments.

### 3.3 CUL1 reduced CSE-induced apoptosis and inflammatory response in mPMECs

**Figure 3:**
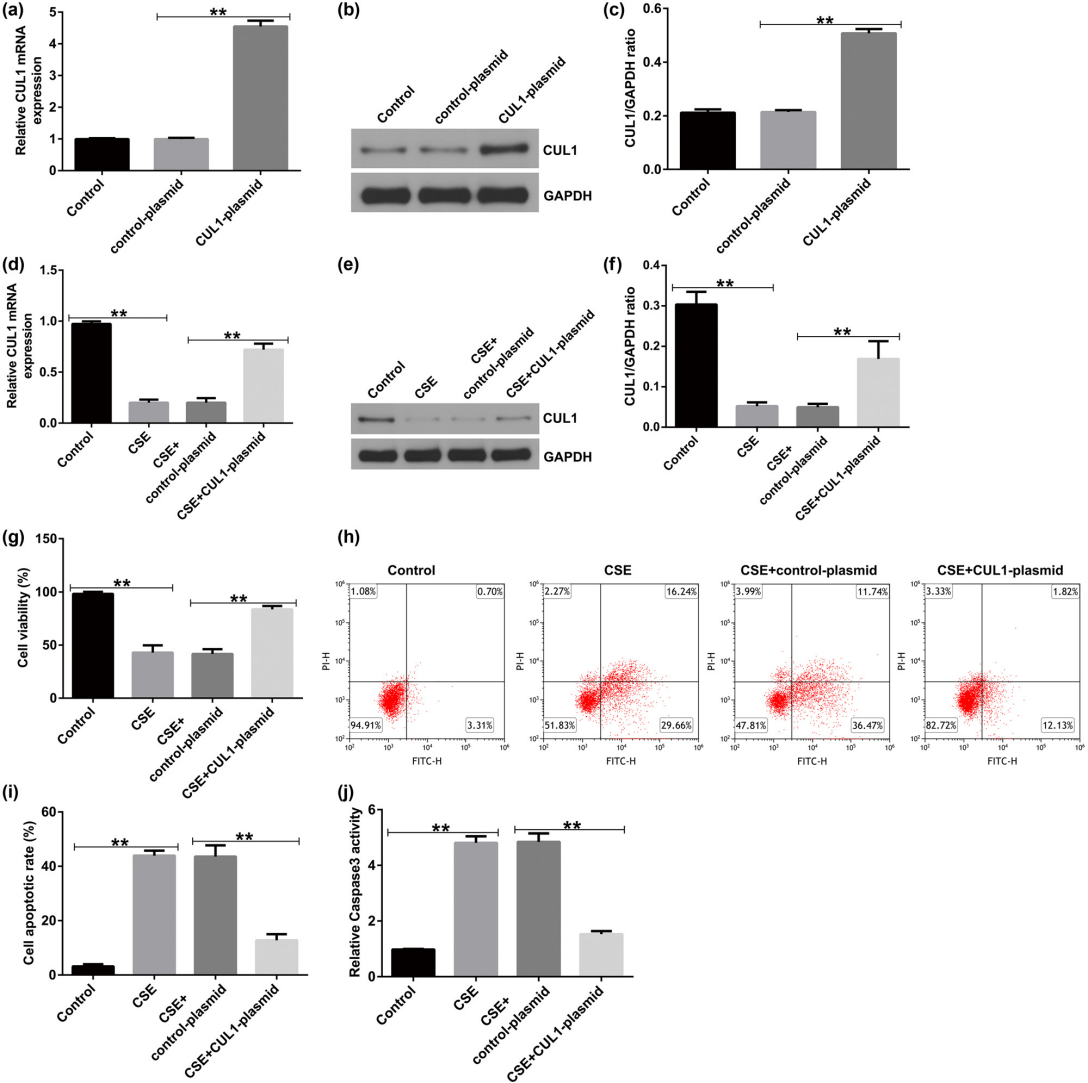
The effect of CUL1 on the apoptosis of CSE-induced mPMECs. (a–c) mRNA and protein expression levels of CUL1 in mPMECs transfected with control-plasmid and CUL1-plasmid. (d–f) CUL1 expression level detected by qRT-PCR and Western blot after CSE treatment. (g) Viability of mPMECs detected by MTT assay. (h and i) Apoptosis of mPMECs detected by flow cytometry assay. (j) Caspase-3 activity in mPMECs. **P < 0.01. CSE, cigarette smoke extract; CUL1, cullin-1; mPMECs, mouse pulmonary microvascular endothelial cells; MTT, 3-(4,5-dimethylthiazol-2-yl)-2,5-diphenyltetrazolium bromide; qRT-PCR, quantitative real-time reverse-transcription PCR.

To detect transfection efficiency, mPMECs were transfected with the control-plasmid and CUL1-plasmid, respectively, and qRT-PCR and Western blot assays were performed 48 h later. The results showed that the CUL1-plasmid significantly increased the expression of CUL1 in mPMECs compared with the control-plasmid group (Figure 3a–c).

Subsequently, mPMECs were transfected with the control-plasmid or CUL1-plasmid for 24 h and then treated with 1% CSE for 24 h to explore the effect of CUL1 on CSE-induced mPMECs. The CSE group had a low expression level of CUL1, but CUL1 in the CSE + CUL1-plasmid group was obviously increased (Figure 3d–f). Cell viability significantly decreased in the CSE group and notably increased in the CSE + CUL1-plasmid group (Figure 3g). Cell apoptosis and caspase-3 activity were significantly increased in the CSE group, whereas the opposite trend was observed in the CSE + CUL1-plasmid group (Figure 3h–j).

**Figure 4:**
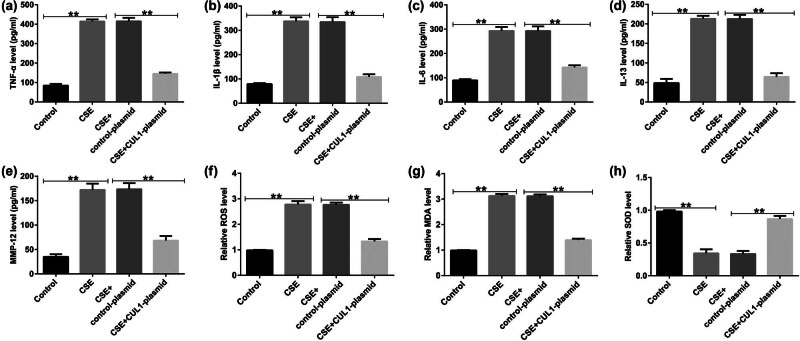
The effects of CUL1 on inflammatory responses and oxidative stress in CSE-induced mPMECs. (a–e) Secretion of TNF-α, IL-1β, IL-6, IL-13, and MMP-12 in mPMECs. (f–h) ROS, MDA, and SOD levels detected by the corresponding kit. **P < 0.01. CSE, cigarette smoke extract; CUL1, cullin-1; IL-1β, interleukin-1β; IL-6, interleukin-6; IL-13, interleukin-13; MDA, malondialdehyde; MMP-12, matrix metalloproteinase-12; mPMECs, mouse pulmonary microvascular endothelial cells; ROS, reactive oxygen species; SOD, superoxide dismutase; TNF-α, tumor necrosis factor-α.

ELISA was used to detect the secretion of inflammatory cytokines. The results showed that the secretion of TNF-α, IL-1β, IL-6, IL-13, and matrix metalloproteinase-12 (MMP-12) was increased in the CSE group but showed a significant decreasing trend in the CSE + control-plasmid group (Figure 4a–e). In the CSE group, the levels of ROS and MDA were observably increased, and SOD activity was decreased, which was the opposite of the pattern observed in the CSE + CUL1-plasmid group (Figure 4f–h). These results revealed that CUL1 can reduce the CSE-induced apoptosis of mPMECs and attenuate the CSE-induced inflammatory response in mPMECs.

### 3.4 CUL1 inhibited the p53 signaling pathway in CSE-induced mPMECs

**Figure 5:**
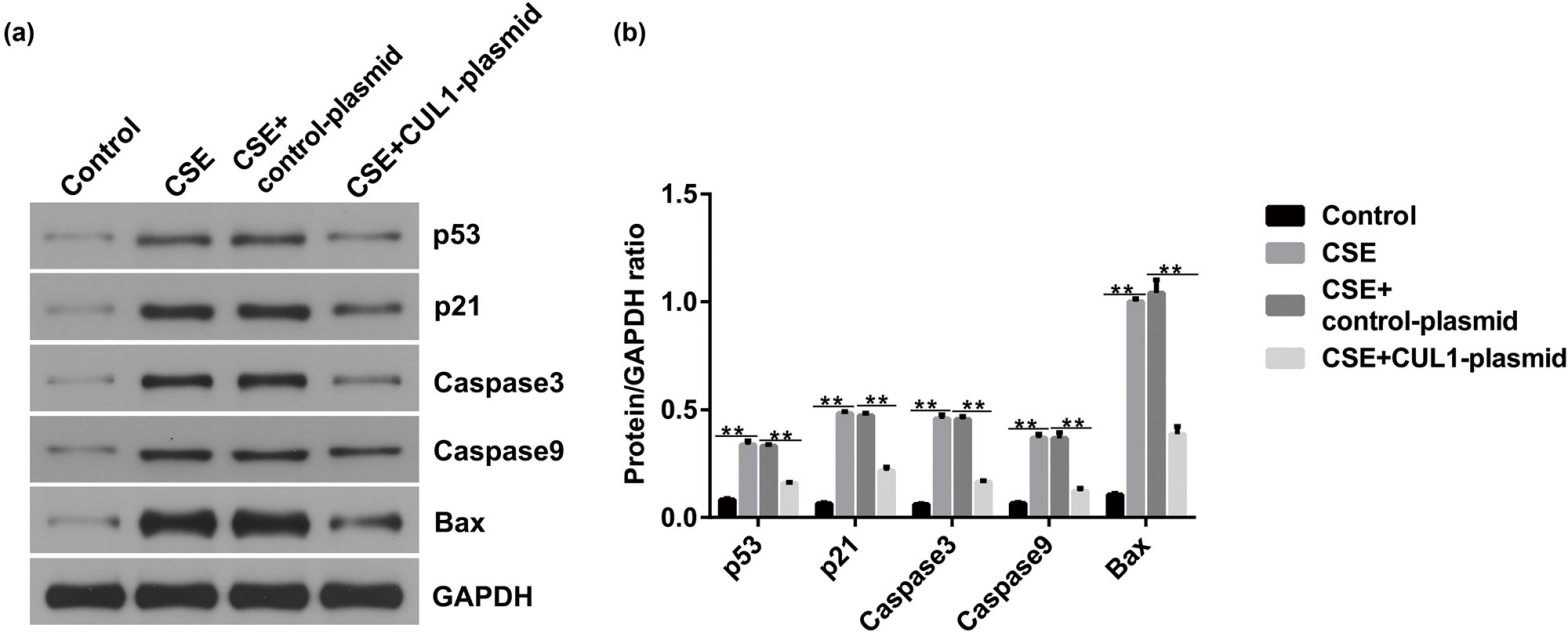
The effect of CUL1 on p53 signaling in CSE-induced mPMECs. (a and b) Protein expression levels of p53, p21, caspase-3, caspase-9, and Bax were detected by Western blot assay with quantitative patterns shown. **P < 0.01. Bax, Bcl-2-associated X protein; CSE, cigarette smoke extract; CUL1, cullin-1; mPMECs, mouse pulmonary microvascular endothelial cells.

To study the molecular mechanisms of CUL1 in CSE-induced mPMECs, we transfected the control-plasmid and CUL1-plasmid into mPMECs for 24 h and then cultured mPMECs with 1% CSE for 24 h. Western blotting showed that the expression of p53, p21, caspase-3, caspase-9, and Bax was significantly increased in the CSE group, and the increased effect was significantly inhibited in the CUL1-plasmid group (Figure 5a and b). Taken together, these results indicate that CUL1 inhibits the p53 signaling pathway in CSE-induced mPMECs.

### 3.5 Nutlin-3 significantly reversed the effect of CUL1 on CSE-induced mPMECs

**Figure 6:**
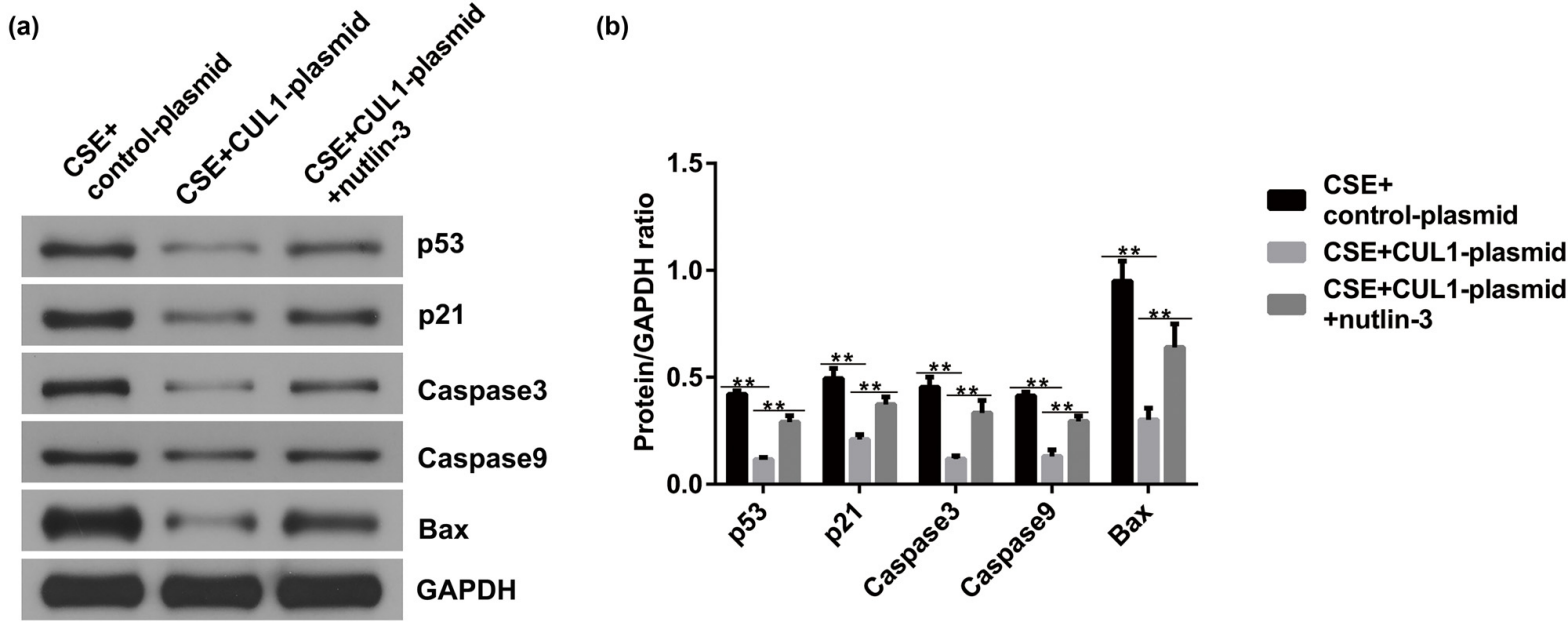
The effects of CUL1 and nutlin-3 on p53 signaling in CSE-induced mPMECs after nutlin-3 treatment. (a and b) Protein expression levels of p53, p21, caspase-3, caspase-9, and Bax detected by Western blot assay with quantitative patterns shown. **P < 0.01. Bax, Bcl-2-associated X protein; CSE, cigarette smoke extract; CUL1, cullin-1; mPMECs, mouse pulmonary microvascular endothelial cells.

To further confirm the role of CUL1 in the p53 signaling pathway in CSE-induced mPMECs, we treated mPMECs with 20 μM of the p53 agonist nutlin-3, and then the control-plasmid and CUL1-plasmid were transfected into the cells for 24 h. After treating the cells with 1% CSE for another 24 h, we performed a Western blot assay. The results showed that the p53, p21, caspase-3, caspase-9, and Bax proteins were expressed at low levels in the CSE + CUL1-plasmid group compared with the CSE + control-plasmid group, but the effect was reversed by nutlin-3 (Figure 6a and b).

**Figure 7:**
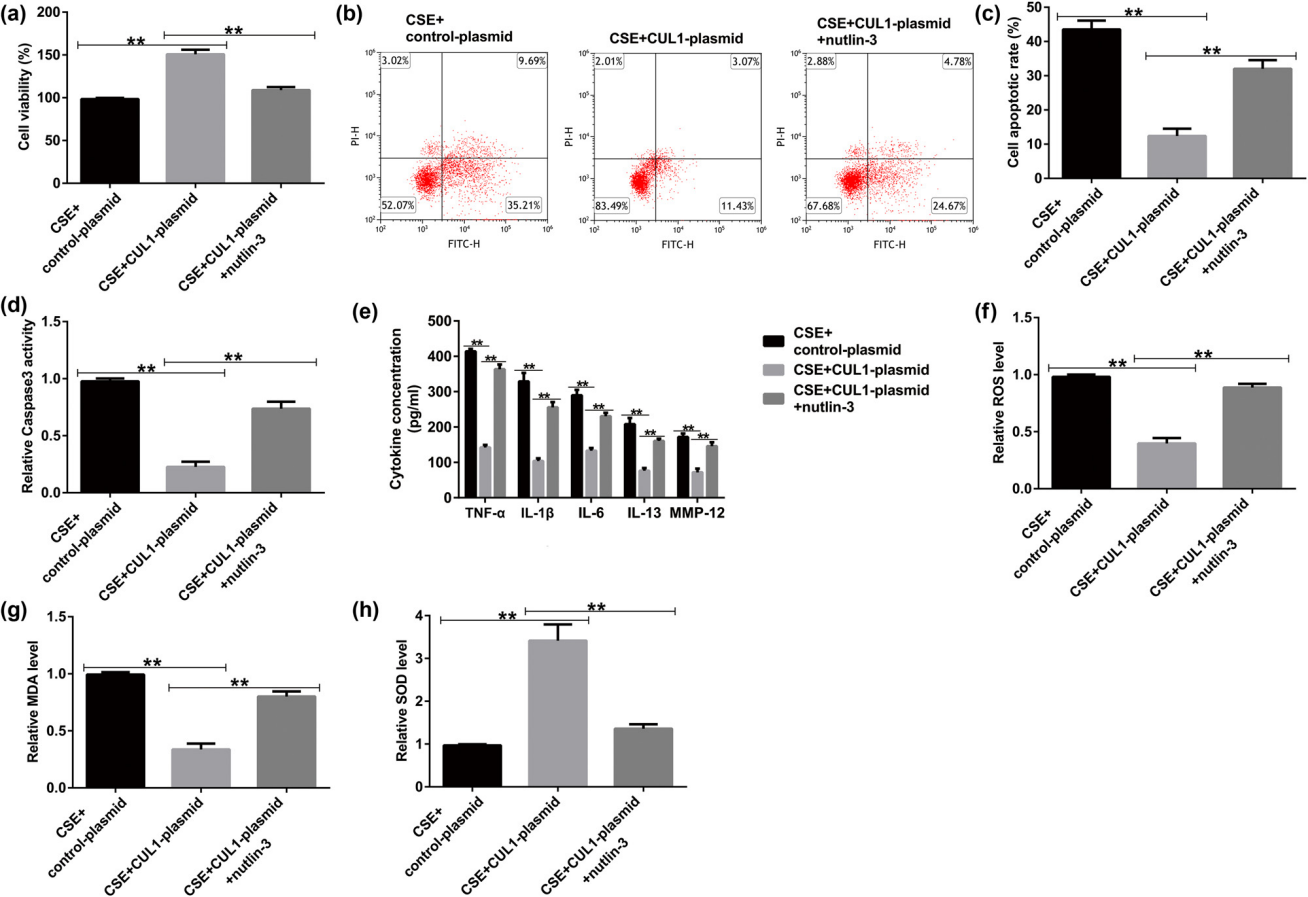
The effects of CUL1 and nutlin-3 on CSE-induced mPMECs. (a) Viability of mPMECs detected by MTT assay. (b and c) Apoptosis of mPMECs detected by flow cytometry assay. (d) Caspase-3 activity in mPMECs. (e) Secretion of TNF-α, IL-1β, IL-6, IL-13, and MMP-12 in mPMECs. (f–h) ROS, MDA, and SOD levels detected by the corresponding kit. **P < 0.01. CSE, cigarette smoke extract; CUL1, cullin-1; IL-1β, interleukin-1β; IL-6, interleukin-6; IL-13, interleukin-13; MDA, malondialdehyde; MMP-12, matrix metalloproteinase-12; mPMECs, mouse pulmonary microvascular endothelial cells; MTT, 3-(4,5-dimethylthiazol-2-yl)-2,5-diphenyltetrazolium bromide; ROS, reactive oxygen species; SOD, superoxide dismutase; TNF-α, tumor necrosis factor-α.

In addition, the MTT assay was used to measure cell viability. The results showed that the cell viability of the CSE + CUL1-plasmid group was significantly improved (Figure 7a), and cell apoptosis was reduced by the CUL1-plasmid (Figure 7b and c). At the same time, caspase-3 activity was significantly decreased in the CSE + CUL1-plasmid group (Figure 7d). However, these effects were reversed upon treatment with nutlin-3.

Upon analyzing the secretion of inflammatory factors after nutlin-3 treatment, we found that the secretion of TNF-α, IL-1β, IL-6, IL-13, and MMP-12 was notably decreased in the CSE + CUL1-plasmid group (Figure 7e), and the same pattern was observed for the levels of ROS and MDA (Figure 7f and g). In addition, SOD activity significantly increased (Figure 7h). However, these effects were reversed upon treatment with nutlin-3. These results show that nutlin-3 reversed the effect of CUL1 on CSE-induced mPMECs, further clarifying the protective effect of CUL1 by inhibiting the p53 signaling pathway.

### 3.6 CUL1 was downregulated in the lung tissues of mice with COPD

**Figure 8:**
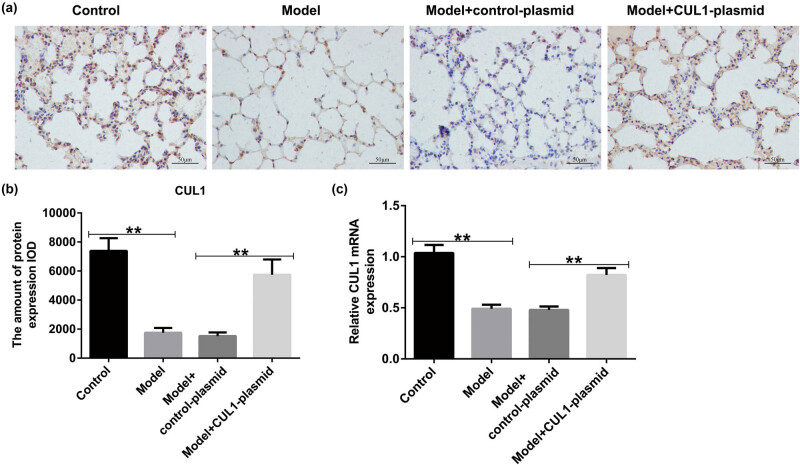
The expression level of CUL1 in lung tissues of mice with COPD. (a and b) IHC staining was conducted to evaluate the distribution of CUL1. (c) mRNA expression level of CUL1 detected by qRT-PCR. **P < 0.01. COPD, chronic obstructive pulmonary disease; CUL1, cullin-1; IHC, immunohistochemistry; qRT-PCR, quantitative real-time reverse-transcription PCR.

To explore the role of CUL1 in the progression of COPD, we generated the COPD mouse model *in vivo* using cigarette smoke. As presented in Figure 8a–c, the expression of CUL1 was reduced in the model group mice, but this inhibition was reversed by treatment with the CUL1-plasmid compared with the control-plasmid group. Our findings strongly suggest that CUL1 is associated with the development of COPD.

### 3.7 Upregulation of CUL1 improved the lung function of mice with COPD

**Figure 9:**
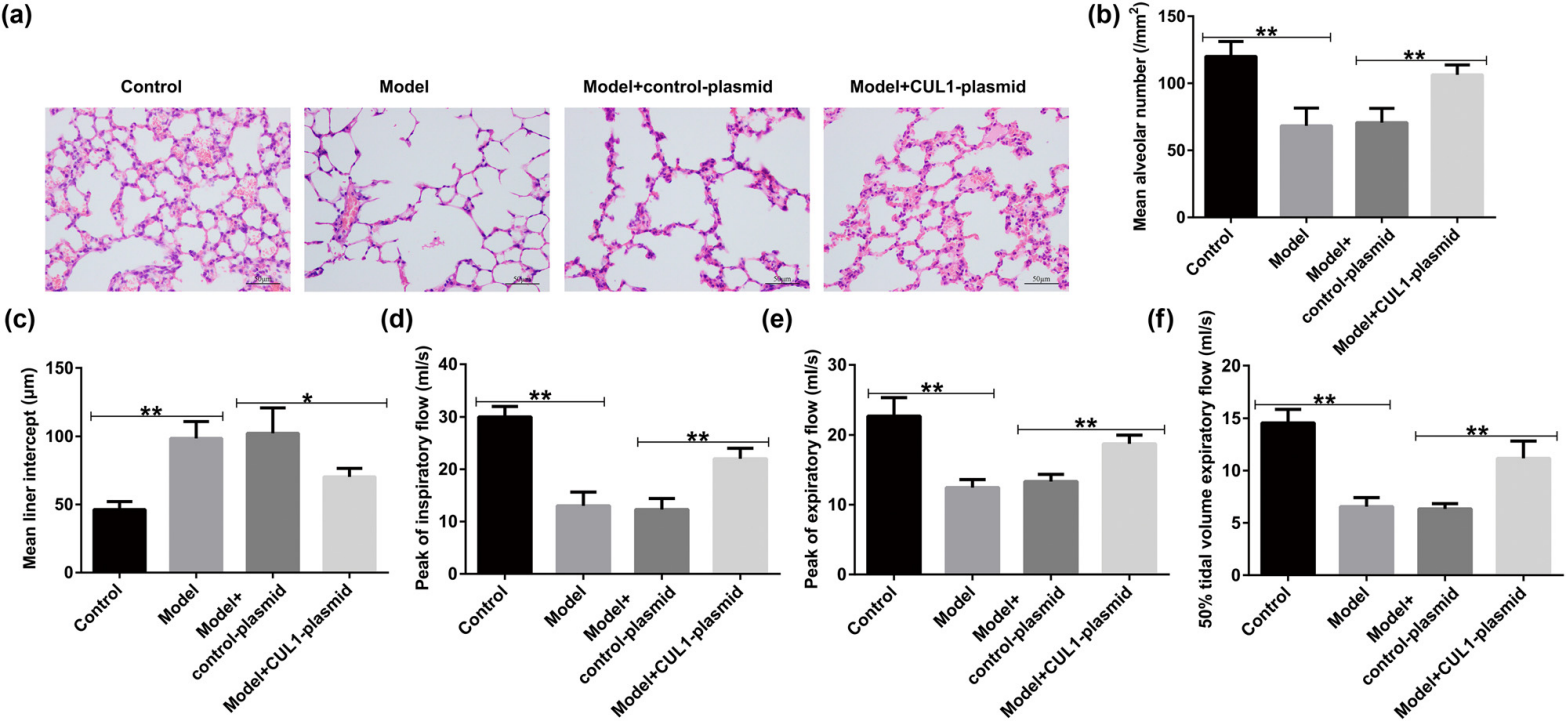
The effects of the CUL1-plasmid on lung function. (a) Pathological changes in the lung tissues of mice detected by H&E staining. (b and c) Changes in MAN and MLI in different groups. (d–f) Calculation of PIF, PEF, and EF_50_ to evaluate the pulmonary function. *P < 0.05, **P < 0.01. CUL1, cullin-1; EF_50_, expiratory flow at 50% ventilation; H&E, hematoxylin and eosin; MAN, mean alveolar number; MLI, mean linear intercept; PEF, peak expiratory flow; PIF, peak inspiratory flow.

We also evaluated the effects of CUL1 on lung function in mice with COPD. According to the results of H&E staining, the lung tissue structure of the mice in the control group was intact, the alveolar size was normal, and the alveolar septum structure was clear. Compared with the control group, the mice in the model group showed severe rupture of the alveolar septum, obvious expansion of the alveolar cavity, and partial alveolar fusion to form pulmonary bullae (Figure 9a–c). However, the lung tissue damage was significantly reduced by CUL1-plasmid intervention, indicating that CUL1 plays a protective role in the pulmonary function of mice with COPD. Compared with the control group, PIF, PEF, and EF_50_ were remarkably reduced in the model group, whereas these parameters were obviously elevated after CUL1-plasmid treatment (Figure 9d–f).

### 3.8 Upregulation of CUL1 suppressed the secretion of pro-inflammatory cytokines and oxidative stress response in the COPD mouse model

**Figure 10:**
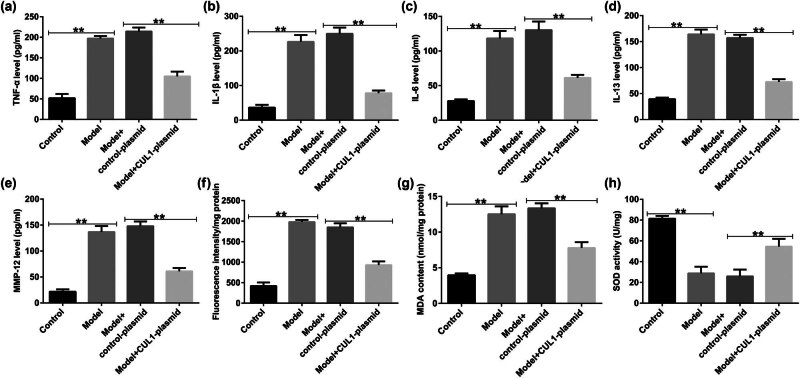
The effects of the CUL1-plasmid on lung tissue inflammation and oxidative stress response. (a–e) Levels of inflammatory factors, such as TNF-α, IL-1β, IL-6, IL-13, and MMP-12, detected in BALF samples using ELISA. (f–h) ROS, MDA, and SOD levels in lung tissues. **P < 0.01. BALF, bronchoalveolar lavage fluid; CUL1, cullin-1; ELISA, enzyme-linked immunosorbent assay; IL-1β, interleukin-1β; IL-6, interleukin-6; IL-13, interleukin-13; MDA, malondialdehyde; MMP-12, matrix metalloproteinase-12; ROS, reactive oxygen species; SOD, superoxide dismutase; TNF-α, tumor necrosis factor-α.

We also focused on the underlying mechanisms of CUL1 in regard to the secretion of inflammatory cytokines and oxidative stress response in mice with COPD. As shown in Figure 10a–e, the contents of TNF-α, IL-1β, IL-6, IL-13, and MMP-12 in the BALF of mice were elevated in cigarette smoke-induced mice compared with the control group. Moreover, the production of ROS and MDA increased, and SOD activity was repressed in the lung tissues of mice with COPD (Figure 10f–h). However, these findings were reversed by the upregulation of CUL1, which demonstrated that CUL1 exerts a protective role in COPD by inhibiting the secretion of inflammatory cytokines and the oxidative stress response.

## 4 Discussion

COPD is a common chronic disease characterized by persistent airflow restriction. This condition is often characterized by coughing, expectoration, and dyspnea, accompanied by a series of complications that lead to chronic systemic inflammation [19]. Currently, in addition to conventional prevention methods (smoking cessation, avoiding exposure to pollution, and adequate exercise) and drug treatment methods (LABAs, LAMAs, and ICS), a series of molecularly targeted therapies have also been studied. Studies have shown that the signal transducer and activator of transcription (STAT) signaling pathway are involved in the pathogenesis of COPD [20]. A pan-Janus kinase (JAK) inhibitor suppressed the increase in phosphorylated STAT 3 (pSTAT3) in the lungs of mice induced by IL-6 *in vivo* after tracheal administration [21]. In addition, several p38α mitogen-activated protein kinase (MAPK) inhibitors can be used to treat COPD, and these drugs significantly reduce the *in vivo* expression of MAPK-driven inflammatory mediators (such as TNF-α and IL-1β) in mouse models of acute inflammation [22]. As smoking is an important risk factor for COPD, many researchers have chosen CSE or CSI to construct cell and animal models for studies on COPD [23,24]. In this study, CSE and CSI were similarly used to treat mPMECs or mice to construct damage models for subsequent studies.

CUL1, together with the F-box protein, adaptor protein Skp1, and RING-box proteins 1 and 2 (RBX1 and RBX2), forms the SCF E3 ubiquitin ligase complex, which affects a variety of biological processes. These include cell growth and development, cell signaling pathways, transcriptional regulation of gene expression, and tumor suppression [25]. CUL1 activity can perform tumor-suppressive functions in cells [26]. In addition, CUL1 overexpression improves the proliferative ability of melanoma cells by regulating the level of p27 [27] and promotes the migration of human trophoblast cells [28]. CUL1 overexpression has also been associated with poor prognosis in gastric cancer, non-small-cell lung cancer, and breast cancer [29,30,31]. Studies have shown that CUL1 is involved in the development of COPD; however, its mechanism of action remains unclear. Therefore, in our study, we investigated this association and found low expression levels of CUL1 in patients with COPD, CSE-induced mPMECs, and CSI-treated mice.

The MTT assay detects the number of viable cells by detecting succinate dehydrogenase in the mitochondria, whereas flow cytometry detects the occurrence of apoptosis by labeling apoptotic cells with annexin V and PI [32]. Similarly, caspase-3 is also used to detect cell apoptosis in view of its role as a key enzyme in the process of apoptosis [33]. ROS, MDA, and SOD activity assays can be used to detect apoptosis by measuring oxidative stress levels [33,34]. Through the above assays, we found that CUL1 suppressed the apoptosis of CSE-induced mPMECs and alleviated the inflammatory response of cells as detected by ELISA.

The p53 signaling pathway is an important regulatory pathway for cell survival and death that can promote DNA repair, prevent mutations caused by DNA damage, maintain genomic stability, induce apoptosis, and protect whole tissues from damaged cells, thereby maintaining normal tissue and organ function [35]. This pathway is involved in the development of cancers such as gastric, pancreatic, and lung cancer [36,37,38]. In addition, CUL1 can inhibit atherosclerosis through the p53 pathway [39]. In our study, CUL1 protected mPMECs from CSE-induced apoptosis and inflammatory responses by inhibiting the p53 signaling pathway. Nutlin-3 is a low-molecular-weight *cis*-imidazoline analog that can induce the regulation and activation of the p53 pathway [40]. Several studies have shown that nutlin-3 can activate the p53 pathway to promote apoptosis without genotoxicity [41,42]. Therefore, in this study, we used nutlin-3 to reverse the effects of CUL1 on CSE-induced mPMECs by activating the p53 signaling pathway, further demonstrating that CUL1 has a protective effect on CSE-damaged cells by inhibiting the p53 signaling pathway.

The primary pathological changes associated with COPD are chronic bronchitis and emphysema [43]. In the present study, we found that rats with cigarette smoke-induced COPD exhibited a significant decline in lung function. In addition, the lung tissues revealed significant histopathological changes, including a reduction in the number of alveoli, narrowing of the alveolar space, partial rupture, and fusion to form bullae, thereby leading to emphysema. A major finding of this study was that CUL1 treatment caused changes in lung function, including a reduction in PIF, PEF, and EF_50_, as well as a significant improvement in lung histopathology, similar to the results of previous studies [44]. Furthermore, the analysis of inflammatory factors in the BALF of mice in each group showed that CUL1 treatment improved the inflammatory response and reduced oxidative damage in mice with COPD. Our findings are consistent with those of a report by Li et al. showing that CUL1 is a vital regulator of the progression of COPD [45].

There were also some limitations of this study. This study did not analyze the correlation between CUL1 expression and clinicopathological parameters in patients with COPD. In addition, whether there are other signaling pathways involved in the effect of CUL1 on COPD remains to be further explored. We will perform these issues in the future.

In summary, our study is the first to report that the upregulation of CUL1 inhibits the development of COPD by reducing the secretion of inflammatory cytokines and inhibiting the oxidative stress response through the p53 signaling pathway, demonstrating that CUL1 may be a potential therapeutic target in COPD. However, the pathogenesis of COPD is complex, and the exact mechanism by which CUL1 suppresses the development of COPD needs further elucidation.
